# Cervical Cancer Cells Express Markers Associated with Immunosurveillance

**DOI:** 10.1155/2019/1242979

**Published:** 2019-05-06

**Authors:** Adriana Gutiérrez-Hoya, Octavio Zerecero-Carreón, Arturo Valle-Mendiola, Martha Moreno-Lafont, Rubén López-Santiago, Benny Weiss-Steider, Isabel Soto-Cruz

**Affiliations:** ^1^Laboratorio de Oncología Molecular, FES Zaragoza, Universidad Nacional Autónoma de México, Batalla 5 de mayo s/n Col. Ejército de Oriente, CP 09230 Ciudad de México, Mexico; ^2^Cátedras CONACyT, CONACYT, Avenida Insurgentes Sur 1582, Benito Juárez, Crédito Constructor, 03940 Ciudad de México, Mexico; ^3^Departamento de Inmunología, Escuela Nacional de Ciencias Biológicas, Instituto Politécnico Nacional, Prolongación de Carpio y Plan de Ayala S/N. Colonia Santos Tomás, Miguel Hidalgo, 11340 Ciudad de México, Mexico

## Abstract

Cervical cancer is the second most frequent cancer in women in Mexico, and its development depends on the presence of human papillomaviruses in the uterine cervix. These oncogenic viruses transform cells where the control over cell cycle disappears, and the capacity to induce apoptosis is absent. On the other hand, some mutations confer to the transformed cells the ability to evade recognition by the immune system. The expression of markers of the immune system such as CD95, MICA/B, CD39, CD73, NKp30, NKp46, CD44, CD24, NKG2A, and CTLA-4 was analysed by flow cytometry on cervical cancer cells INBL (HPV 18, stage IVB), HeLa (HPV 18), CaSki (HPV 16), and C33A (HPV-). Our results showed the presence of atypical markers on cervical cancer cells; some of them are molecules involved in tumour cell recognition such as MICA/B and CD95. Other markers associated with immune system escape, such as CD39, CD73, and CTLA-4, were also present. Furthermore, we found that some cervical cancer cells expressed typical markers of NK cells like NKp30, NKp46, NKG2A, and KIR3DL1. It is not clear whether these molecules confer any gain to the tumour cells or if they represent a disadvantage, but we hypothesise that these molecules that are present in cervical cancer cells allow them to mimic in front of the immune system.

## 1. Introduction

One of the main strategies of the transformed cells to establish premalignant and malignant lesions is the modulation of the microenvironment and the evasion of the immune system. Our research group determined that cervical cancer cells express diverse molecules that play a central role in cell proliferation and that could serve as biomarkers of the disease. One of these molecules is the IL-2 receptor (IL-2R) which is present in cervical cancer cells. These cells express the *α*, *β*, and *γ* subunits of IL-2R; the addition of exogenous IL-2 induces the interaction with its receptor to initiate the JAK/STAT signalling pathway essential for proliferation, similar to that activated in lymphocytes [1, 2]. These tumour cells also express constitutively active JAK3 and STAT5 proteins [3]. Another set of molecules involved in tumour cell growth are proteins that belong to the epidermal growth factor receptor (EGFR) family. Data from our working group show that EGFR and HER2 are also present in cervical cancer lines and that they could be involved in the pathogenesis and progression of cervical lesions, due to a direct association of EGFR expression and HPV infection [4]. However, tumour cells are also able of expressing atypical receptors along with their respective ligands to induce tumour proliferation. For example, a small subpopulation of cervical cancer cells express the NKG2D receptor on the cell surface and secrete the ligands MICA/B and thereby induce tumour proliferation and probably use these molecules as an immunological escape mechanism [5]. The presence of these tumour markers that can be used by cervical cancer cells to induce their proliferation or, furthermore, an evasion of the immune system, led us to evaluate the presence of other typical and atypical cell markers to elucidate their role in the development of cervical cancer and consider their use as suitable tumour markers.

## 2. Materials and Methods

### 2.1. Cell Lines and Antibodies

Biological materials, the HeLa (HPV 18), CaSki (HPV 16), and C33A (HPV-) cell lines, were obtained from the American Type Culture Collection (ATCC). INBL, an HPV 18 cell line derived from an invasive stage IVB squamous cell carcinoma, was established at the Cell Differentiation Laboratory of FES Zaragoza [6]. Cells were cultured in RPMI-1640 medium (Microlab, Mexico) supplemented with 5% foetal bovine serum (FBS, Invitrogen, USA). All cultures were maintained in an incubator at 37°C, 5% CO_2_, and saturated humidity.

### 2.2. Analysis of Cell Surface and Intracellular Markers of Tumor Cells

Approximately 1 × 10^6^ cervical cancer cells per condition were seeded onto Petri dishes. Cells were incubated for 35 min at 4°C in staining buffer (PBS, 0.5% BSA, and 0.01% sodium azide). The cells were stained with specific antibodies against the following surface markers: anti-NKp30-PE, anti-CD95-APC, anti-CD39-FITC, anti-CD-73-PE-Cy7, anti-KIR3DL1-APC, anti-NKp46-APC, anti-CD25-PE, anti-CD25-PerCP-Cy5.5, anti-CD44-FITC, and anti-CD24-PE (Becton Dickinson, USA); anti-NKG2A-PerCP, anti-CD158e1-APC (R&D, Minneapolis, MN); and anti-CD158b2-FITC (BioLegend). Cells were washed and fixed with 1% paraformaldehyde for 20 minutes. For permeabilisation, cells were treated with Cytofix/Cytoperm buffer for 20 minutes (Becton Dickinson, USA). For intracellular staining, anti-human CTLA-4-APC, anti-CD95-PE-Cy7 (Becton Dickinson, USA), and anti-NKG2A-PerCP (R&D, Minneapolis, MN) antibodies were used. Cell samples were incubated for 35 minutes at 4°C in the dark. Finally, cells were washed and resuspended in staining buffer. The phenotype of tumor cells was characterized by flow cytometry on a FACSAria II cytometer (Becton Dickinson, USA). Data were analysed using Summit 4.4 software.

## 3. Results and Discussion

### 3.1. Activating Ligands of the Immune Response Are Differentially Expressed by Cervical Cancer Cell Lines

**Figure 1:**
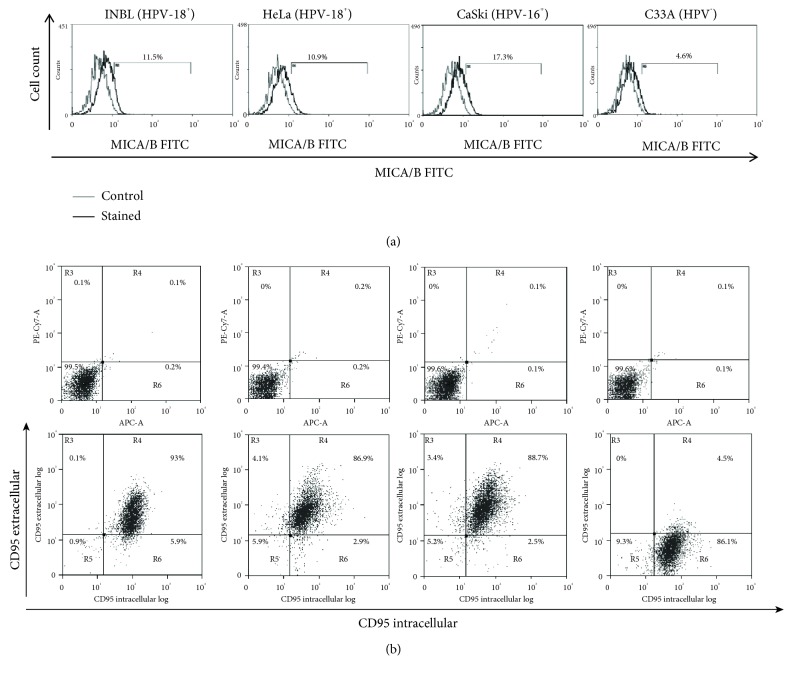
Presence of activating markers of the immune response in cervical cancer cells. (a) Expression of MICA/B on the INBL, HeLa, CaSki, and C33A cells was determined using anti-MICA/B antibodies conjugated with FITC. In the histograms, the grey lines represent the staining controls and the black lines represent the stained cells. (b) Expression of CD95 extracellular and intracellular on the INBL, HeLa, CaSki, and C33A cells was determined using anti-CD95 antibodies conjugated with PE-Cy7 (for extracellular expression) and APC (for intracellular expression); the upper dot plots show the staining controls for each cell line, and the lower dot plots show the cells stained for CD95.

We analysed the expression of MICA/B and CD95, markers of the immune system, in cervical cancer cell lines infected with HPV18 (INBL and HeLa), HPV16 (CaSki), and HPV-negative (C33A). MICA or MICB is not expressed on the cell surface of healthy cells; these molecules are known to activate cytotoxic cells, including NK cells and CD8 T cells. We previously demonstrated that cervical cancer cells express the NKG2D receptor on the surface membrane, and the interaction of NKG2D with MICA/B induced the proliferation on tumour cells [5]. Our results indicate that MICA/B expression in cervical cancer cells is low (4-17%, Figure 1(a)); however, it is present in all cell lines. These results are consistent with those of other research groups that demonstrated that MICA or MICB is present in most tumour samples analysed, with predominantly intracellular localisation and only occasional cell surface localisation. Nevertheless, they report a similar profile for many epithelial cells in normal tissues [7]. It is not clear why normal tissues do express MICA or MICB, but our results with cervical cancer cells suggest that it is a strategy to evade the immune system. Our workgroup demonstrated that a small subpopulation of the cervical cancer cell lines bears the NKG2D receptor, and by interacting with its ligands (MICA/B), tumour proliferation is induced [5]. Thus, the presence of these molecules might help tumour cells to proliferate and evade the immune system.

CD95 (Fas/APO-1) and its ligand, CD95L, are very important for inducing apoptosis to maintain immune homeostasis and for the elimination of virus-infected cells, damaged cells, and cancer cells. We analysed whether the cervical cancer cells expressed CD95 on the cell membrane, and our results demonstrated that cervical cancer cells positive for HPV (INBL, HeLa, and CaSki) show high levels of CD95 (86-93%). C33A cells, negative for HPV, express shallow levels of CD95 (4.5%; Figure 1(b)). To further explore the presence of CD95 in C33A cells, we evaluated the presence of intracellular CD95. Our results indicate that all cervical cancer cell lines, including C33A, express high intracellular levels of CD95 (90-99%; Figure 1(b)). Filippova et al. showed that the E6 protein of HPV-16 could bind to the death domains of FAS (FADD) and thereby degrade them. The loss of FADD prevents the activation of the apoptotic cascade and with this the elimination of the tumour cells [8]. Additional experimental studies have referred to the presence of CD95L with a poor prognosis in cervical cancer [9, 10], which was initially attributed as a mechanism for immune escape. Recently, some studies report that several tumour cells can use the CD95/CD95L pathway to induce survival and proliferation following the activation of signalling pathways such as NF-*κ*B, Erk1/2, JNK, and Jun [11]. Altogether, results suggest that CD95 could be considered as an important marker that might be involved in tumour growth and survival rather than in tumor elimination.

### 3.2. Inhibitory Markers of the Immune Response Expressed by Cervical Cancer Cell Lines

**Figure 2:**
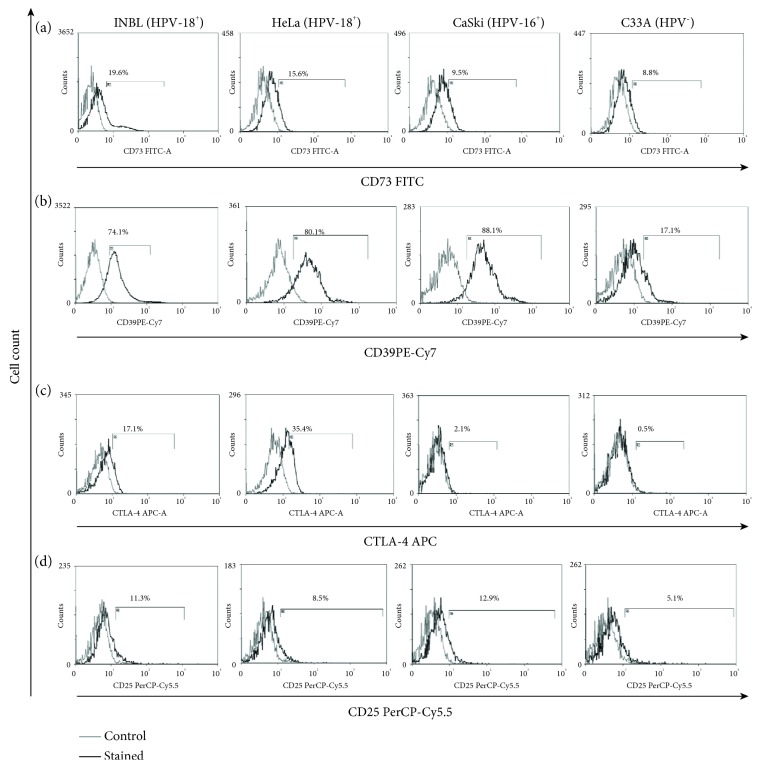
Inhibitory markers of the immune response present in cervical cancer cells. (a) The expression of CD73 on INBL, HeLa, CaSki, and C33A cells. (b) The expression of CD39 on INBL, HeLa, CaSki, and C33A cells. (c) The expression of CTLA-4 on INBL, HeLa, CaSki, and C33A cells. (d) The expression of CD25 on INBL, HeLa, CaSki, and C33A cells. In the histograms, the grey lines represent the staining controls and the black represent the stained cells.

Tumour cells have different strategies to evade the immune system; one strategy is the secretion of immunomodulatory cytokines such as IL-10 and TGF*β*. Another approach is the expression of molecules that suppress the immune response such as CTLA-4, CD25, CD39, and CD73. These molecules are important markers in Treg cells and contribute by inhibiting the activation of dendritic cells by joining CTLA-4/CD80 and CD86 and by deprivation of ATP, a role played by the CD39 and CD73 ectonucleases or for the elimination of IL-2 by CD25. CD39 hydrolyses extracellular ATP and adenosine diphosphate (ADP) into adenosine monophosphate (AMP), and then AMP is degraded into adenosine by the CD73 ecto-5′-nucleotidase. Finally, this adenosine binds to A2A receptors. Adenosine causes the accumulation of intracellular cAMP preventing TCR-triggered CD25 upregulation and inhibiting activation of T lymphocyte proliferation and the secretion of inflammatory cytokines [12]. We evaluated the presence of markers that suppress the immune response such as CD73 and CD39. The results showed that cervical cancer cells express CD73 and CD39 (Figures 2(a) and 2(b)). These data are consistent with the expression of CD73 which plays a significant role in the production of adenosine that suppresses antitumour effector cells, as well as with the fact that cervical cancer cells that are HPV-positive express a higher percentage suggesting that HPV infection contributes to overexpression of CD73 [13]. However, the role of CD39 in cervical cancer is poorly understood. Studies have shown that tumour cells like B lymphoma cells, melanoma cells, B cell chronic lymphocytic leukaemia cells, and ovarian cancer cells overexpress CD39 and thus activate the adenosinergic pathway to inhibit the proliferation of CD4 and CD8 T cells and the cytotoxic effector CD8 T cells in a CD39 adenosine-dependent manner [14]. These data suggest that overexpression of CD39 in cervical cancer cell lines is important for tumour survival. We observed that the expression of CD39 is higher in HPV-positive cells (74-88%) versus HPV-negative cervical tumour cells (17%); this observation points out the importance of the presence of the virus to favour the expression of this molecule.

CTLA-4 plays a crucial role in the regulation of the immune system. We determined its presence on the cell surface of cervical cancer cell lines. The results showed a very low or null presence of CTLA-4 on the cell surface (data not shown). Therefore, we evaluated the intracellular form of this protein. The results revealed that all cervical cell lines express CTLA-4 (0.5-35%; Figure 2(c)). These results are similar to those reported by Contardi et al., who observed a weak presence of CTLA-4 in HeLa and C33A cells; they also evaluated the presence of this molecule in a large number of cell lines derived from a variety of malignant human solid tumours including breast carcinoma, melanoma, neuroblastoma, rhabdomyosarcoma, and osteosarcoma. This working group found that the treatment of cells expressing CTLA-4 with recombinant forms of the costimulatory molecules CD80 and CD86 induced apoptosis associated with the activation of caspase-8 and caspase-3 [15]. However, Chen et al. showed that the coculture of dendritic cells activated with breast cancer cells positive for CTLA-4 decreases the expression of costimulatory molecules. Moreover, the suppressed dendritic cells further inhibited proliferation of CD4^+^/CD8^+^ T cells, which implies the role of CTLA-4 in the immunosuppression of the immune system [16]. We demonstrated the presence of these molecules; however, at this point, the role of CTLA-4 in cervical cancer cells remains to be determined. Additionally, further research is needed to conclude whether these inhibitory markers of the immune response are important in the tumour microenvironment.

CD25 is a critical immunoregulatory molecule constitutively expressed on Treg cells for deprivation of IL-2 to induce apoptosis and suppress effector T cells [17]. CD25, the *α*-chain of IL-2R, is essential to form the high-affinity receptor (*Kd* = 10−11 M) [18]. Previously, our workgroup demonstrated that some HPV 18^+^ cervical cancer cells express the *β* and *γ* chain of the IL-2 receptor [1, 2]. We evaluated the presence of CD25 in HPV-positive and HPV-negative cervical cancer cells; the results showed a low percentage of CD25-positive cells (5-12%; Figure 2(d)). The role of these molecules remains elusive; however, several reports exist about the role of Treg associated with a prognosis in cancer. Nevertheless, only Loose et al. report the expression of CD25 in squamous cell carcinoma of the head and neck (SCCHN), but they did not find any relationship between the CD25 expression on tumour cells with a prognosis in SCCHN patients [19]. We do not know if the presence of this marker confers the tumour cells a strategy to evade the immune response due to IL-2 depletion. But previous reports of our work group show that low doses of IL-2 can be used by cervical tumour cells to proliferate [3].

### 3.3. Epithelial-Mesenchymal Transition Markers or Cancer Stem Cell Population

**Figure 3:**
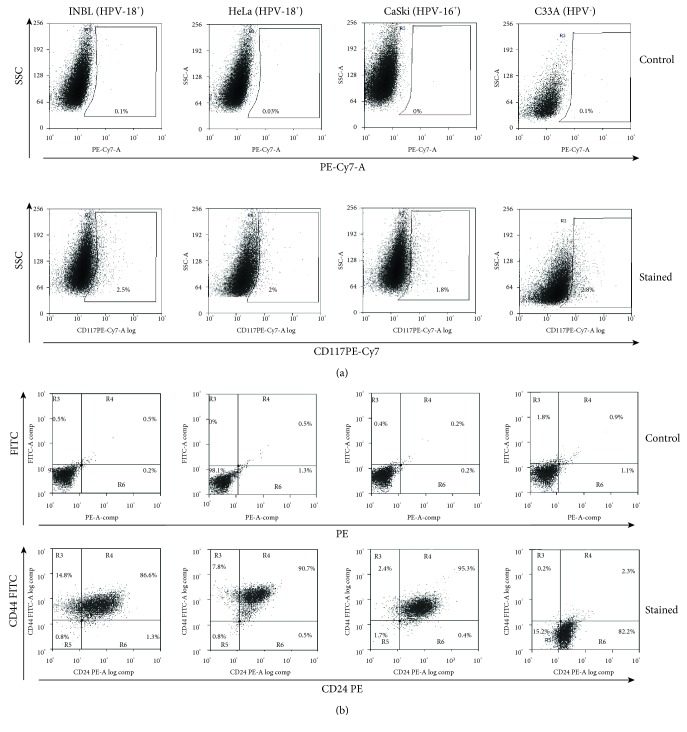
Markers of stem cells and epithelial-mesenchymal transition present in cervical cancer cells. (a) Expression of CD117 (c-kit) on INBL, HeLa, CaSki, and C33A cells was determined using anti-CD117 antibodies conjugated with PE-Cy7. The upper graphics indicating granularity versus fluorescence in PE-Cy7 show the staining controls for each cell line, and the lower graphics indicating granularity versus CD117 PE-Cy7 show the positive cells stained for CD117. (b) Expression of CD24 and CD44 on the INBL, HeLa, CaSki, and C33A cells was determined using anti-CD24 antibodies conjugated with PE and anti-CD44 antibodies conjugated with FITC. The upper dot plots show the staining controls for each cell line, and the lower dot plots show the double-positive cells stained for CD24 and CD44. For C33A cells, only CD24-positive cells were found.

Metastasis is the primary cause of death in patients with cervical cancer; evidence exists that metastasis develops due to the presence of tumour stem cells, which show slow replication, are resistant to chemotherapy, and have a metastatic capacity. One potential marker for the cancer stem-like cell subpopulation is CD117/c-kit, a tyrosine kinase receptor associated with cancer progression and stem cell maintenance. Stimulation of CD117 by its ligand, stem cell factor (SCF; kit ligand), activates several signalling pathways driving proliferation, survival, and migration [20]. The importance of c-kit is evident in renal carcinoma, ovarian cancer, melanoma, acute myeloid leukaemia, and gastrointestinal tumour amongst others [21, 22]. Therefore, we decided to evaluate the presence of c-kit in cervical cancer cell lines. The results showed that only a small proportion of the cells express this marker (1.8-2.9%; Figure 3(a)), but this percentage is similar in cells positive for HVP 18 and 16 or negative for HPV; however, these results suggest that they could represent a subpopulation of cancer stem cells (CSC). Other markers associated with stem cells and the epithelial-mesenchymal transition (EMT) are CD44 and CD24; these markers have been best characterized in breast cancer, where the subpopulation of CSC expresses low levels of stable heat antigen (CD24) and high levels of the hyaluronic receptor (CD44). This cell subpopulation has the capacity of self-renewal, to initiate tumour formation, and is intrinsically resistant to chemotherapy. However, although these markers associate with metastasis, they are not sufficient to predict the capacity of metastasis [23, 24]. On the other hand, Liu et al. showed that HPV16-positive cervical cancer cells SiHa CD44^+^/CD24^+^ are resistant to cellular apoptosis induced by irradiation therapy and that these cells possess characteristics of stem cells such as potent tumorigenicity and colony formation capacity and have high *in vitro* balling ability in suspension culture [25]. We determined the expression of CD44 and CD24 in the different cervical cancer cell lines; the results demonstrate that HeLa, INBL, and CaSki cells express high levels of both molecules (86-95%). However, HPV-negative C33A cells do not express CD44, but they do express CD24 by more than 80% (Figure 3(b)). Taking these results into consideration, we hypothesise that the presence of the virus may control the overexpression of CD44.

A large number of cancers overexpress CD44; however, its role in cervical cancer is unknown. CD44 has a pleiotropic role and is involved in the induction of EMT altering the cytoskeleton, promoting drug resistance, and activating antiapoptotic mechanisms. At present, anti-CD44 antibodies are evaluated in preclinical trials to know if blocking this molecule could serve as a therapy for cancer [26].

### 3.4. NK Cell Markers in Cervical Cancer Cells

**Figure 4:**
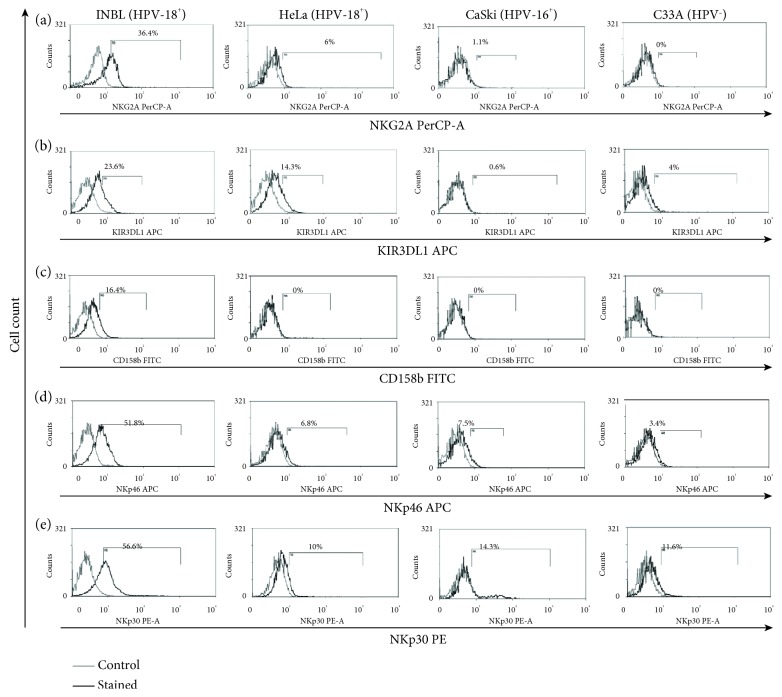
Cervical cancer cells express NK cell markers. (a) Intracellular expression of NKG2A on INBL, HeLa, CaSki, and C33A cells. (b) Expression of KIR3DL1 on INBL, HeLa, CaSki, and C33A cells. (c) Expression of NKp46 on INBL, HeLa, CaSki, and C33A cells. (d) Expression of NKp30 on INBL, HeLa, CaSki, and C33A cells. (e) Expression of CD158b on INBL, HeLa, CaSki, and C33A cells. In the histograms, the grey lines represent the staining controls and the black lines represent the stained cells.

We previously reported that a small subpopulation of cervical cancer cells expresses the NKG2D receptor, a receptor believed to be exclusive of cytotoxic cells. Also, we demonstrated that tumour cells secreted NKG2D ligands MICA/B to induce their proliferation [5]. Later, Tang and collaborators found that NKG2D expression in tumour cells contributes to the immunological escape by the acute myeloid leukaemia cells [27]. With this information in mind, we evaluated the presence of molecules, which are characteristic of cytotoxic cells, in cervical cancer cells. Interestingly, we found that small subpopulations of tumour cells expressed atypical markers, for example, in all HPV-positive cervical cancer cells, we found a small subpopulation that expresses intracellular NKG2A (1-6%). However, a bigger subpopulation (36.4%) of the INBL cell line expresses the NKG2A receptor (Figure 4(a)). The NKG2A receptor plays a vital role in immunosurveillance by binding to HLA-E complexes; it is an inhibitory receptor signalling through two inhibitory ITIM motifs [28]. Some studies showed that blocking the interaction between NKG2A and HLA-E (NKG2A ligand) can regain the activity of NK cells, suggesting that NKG2A blockade has the potential to restore immunity against tumours [29, 30]. At this point, the function of NKG2A in cancer cells is not clear, but in cervical cancer cells, the intracellular expression is high. One possible explanation is that tumour cells could secrete NKG2A to interact with HLA-E expressed by the same tumour cells, and this could activate a survival signal. It is also possible that soluble NKG2A could interact with HLA-A-expressing cells in the immune system blocking their activation; however, this remains to be determined. After evidencing the presence of NKG2A, a molecule with inhibitory functions typically expressed by NK cells, we search for other molecules that interfere with the activation of NK cells. As shown in Figure 4(b), approximately 23% of INBL and 14% of HeLa cells express the inhibitory molecule KIR3DL1, whereas HeLa, CaSki, and C33A express a low percentage of this molecule. The presence of KIR3DL1 on NK cells inhibits the lysis of target cells bearing HLA-Bw4. Also, small populations of *γδ* T cells, CD4^+^ T cells, and CD8^+^ T cells express KIR3DL1 to control its cytolytic ability, cytokine production, and proliferation. Interestingly, Chwae and coworkers showed that the expression of KIR3DL1 could protect these cells from activation-induced cell death following stimulation, at least in part by preventing the upregulation of the Fas ligand [31, 32]. In this context, our results show that HPV-positive cervical cancer cells express a high percentage of CD95 and INBL cells express KIR3DL1; therefore, it is possible that these molecules could inhibit the induction of apoptosis by the CD95 pathway. We evaluated the expression of CD158b (KIR2DL2/DL3) with typically inhibitory functions expressed by NK cells [33]. There are no references on the appearance of this molecule in tumour cells, and there are few references on its expression on cells of the immune system and its association with cancer. Guerra et al. showed the presence of this molecule in infiltrates of lymphocytes in renal carcinomas and how the lythic activity of autologous tumour cells increased with the addition of the anti-CD158b antibody, indicating that this molecule participates in the tumour immune response [34]. Our results show that INBL cells express CD158b (16%; Figure 4(c)), while in the other cell lines, this molecule is not present. We do not know the advantages conferred by the expression of CD158b to these tumour cells, and further research is necessary to analyse if the expression of KIR favours the evasion of the immune system.

To further evaluate the presence of activating markers of the immune system in cervical cancer cells, we analysed whether tumour cells express the activating receptors of NK cells. These receptors belong to the family of Ig-like molecules named natural cytotoxicity receptors (NCRs) which include NKp30, NKp44, and NKp46 molecules. We evaluated the expression of NKp30 and NKp46 in cervical cancer cells. As shown in Figure 4(d), INBL cells express NKp46 (51%) while a low percentage of HeLa (6.8%), CaSki (7.5%), and C33A (3.4%) express this molecule. NKp46 is a specific NK killer receptor that recognises some influenza hemagglutinins and unknown tumour ligands. Cagnano and coworkers showed that malignant melanocytes but not normal melanocytes express ligands to NKp46; these data are consistent with those demonstrated by Elboim et al. where tumour growth of cells expressing high levels of the NKp46 ligands depends upon the presence of the NKp46 receptor [35, 36]. Altogether, these results suggest that tumour cells expressing NKp46 could secrete ligands for this receptor and thereby induce tumour growth. Further research is needed to describe if the stimulation of NKp46-positive cervical tumour cells with its ligand could induce its survival or proliferation.

Finally, we determined the presence of the receptor NKp30, an activator of the immune response in NK cells. Our results show that all cervical cancer cell lines express this receptor; however, INBL cells express a higher percentage of this molecule (56%; Figure 4(e)) while HeLa, CasKi, and C33A express a lower percentage (10-14.3%). NKp30, a natural cytotoxicity receptor on NK cells, is involved in multiple processes, such as allowing NK cells to interact with dendritic cells, modulating the maturation or elimination of dendritic cells, and the maturation of NK cells [37]. One of the ligands for NKp30 is galectin-3; interestingly, cervical tumour cells secrete different galectins, including galectin-3, which participates in the induction of proliferation, metastasis, angiogenesis, and resistance to chemo/radiotherapy. Galectin-3 is associated with the inhibition of apoptosis and the immune response. The concentration of galectin-3 is related to the stage of cervical cancer [38–40]. Since we observed that NKp30 is present in all cervical cancer cell lines, and higher expression in INBL cells, we propose that tumour cells can produce galectin-3 to interact with the NKp30 receptor and maybe promote metastasis, proliferation, angiogenesis, and chemoresistance. However, this requires further investigation to analyse the function of this signalling pathway in tumour cells.

**Figure 5:**
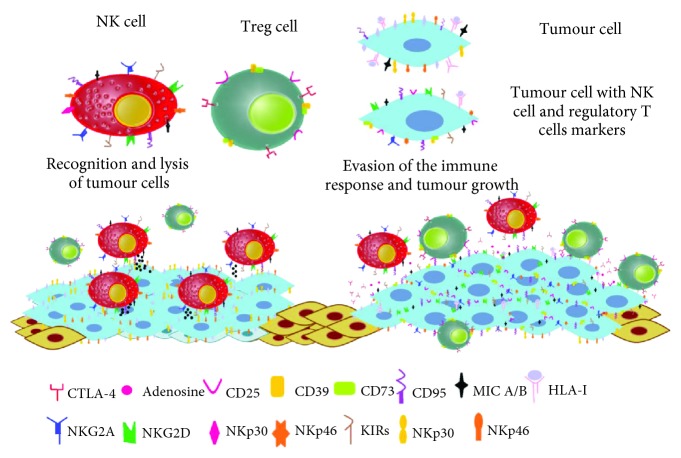
Cancer cells evade the immune system. The tumour cells express markers of the immune system such as CD25, CTLA-4, CD39, and CD73 that help to regulate the tumour microenvironment. Also, these cells express typical markers of NK cells, apparently as a camouflage system to counteract the effect of increased expression of molecules that induce cytotoxicity such as CD95.

Our results show that there is a large variety of subpopulations of tumour cells (Figure 5), some that bear activating markers of the immune response. Evidence exists that cervical tumour cells have strategies to evade the activation of the immune response; thus, it is possible that these molecules instead of activating the immune response promote proliferation, survival, and metastasis. Another set of tumour cells expressing markers of the immune response induce tolerance or lack of activation of the immune system; also, there are small subpopulations that express stem cell and epithelial-mesenchymal transition markers. However, these small subpopulations of tumour cells could be responsible for relapses and metastases. Finally, in this article, we show the presence of typical NK cell markers, molecules that in NK cells promote their inhibition or activation; however, at this point, the effect of these markers in tumour cells is not clear. Further research is needed to conclude whether these molecules contribute to tumour proliferation or to the promotion of metastasis and, furthermore, to determine if tumour cells use these molecules as camouflage to prevent the activation of the immune system.

The analysis of all these molecules provides a better understanding of tumour cell biology and opens the possibility of analysing more signalling pathways related to proliferation, metastasis, or resistance to cell death that could be therapeutic targets. Then, it is necessary to evaluate the presence of each of these molecules in early lesions of the cervix, carcinoma in situ, and invasive cancer, to know whether they have clinical and medical relevance and, further, to determine if they can be functional biomarkers.

## 4. Conclusion

The tumour cells have developed a series of mechanisms that allow them to express molecules that promote their proliferation and enhance its survival capacity. Some of these molecules are typical of their cell lineage, and others are not present in normal tissues of the same origin. Our data indicate that the cervical tumour cells express markers associated with activation of the immune system (CD95 and MICA/B), as well as markers related to inhibition of the immune system (CD73, CD39, CTLA-4, and CD25). However, there are very small subpopulations that express stem cell and epithelial-mesenchymal transition markers. Additionally, some subpopulations of cervical tumour cells express typical receptors of NK cells, activating inhibitor markers. At this point, we do not know if these molecules allow cervical cancer cells to proliferate and enhance its survival capacity. Altogether, our results suggest that these molecules could provide cervical cancer cells with a useful strategy to mimic the immune cell and therefore the ability to evade the immune response for the development of tumour masses.

## Data Availability

All data obtained during the present study are available from the corresponding author upon reasonable request.
